# Rochester’s Lead Law: Evaluation of a Local Environmental Health Policy Innovation

**DOI:** 10.1289/ehp.1103606

**Published:** 2011-10-14

**Authors:** Katrina Smith Korfmacher, Maria Ayoob, Rebecca Morley

**Affiliations:** 1 Environmental Health Sciences Center, University of Rochester Medical Center, Rochester, New York, USA; 2 Center for Governmental Research, Albany, New York, USA; 3 National Center for Healthy Housing, Columbia, Maryland, USA

**Keywords:** childhood lead poisoning prevention, environmental health policy, healthy housing, local government

## Abstract

Background: Significant progress has been made in reducing the incidence of childhood lead poisoning in the United States in the past three decades. However, the prevalence of elevated blood lead in children (≥ 10 μg/dL) remains high in some communities, particularly those with high proportions of pre-1978 housing in poor condition. Increasingly, municipalities are using local policy tools to reduce lead poisoning in high-risk areas, but little is known about the effectiveness of such policies.

Objectives: In this article, we evaluated the effectiveness of a comprehensive rental housing–based lead law adopted in Rochester, New York, in 2005.

Methods: This policy evaluation integrates analyses of city inspections data, a survey of landlords, landlord focus groups, and health department data on children’s blood lead levels from the first 4 years of implementation of the 2005 law.

Results: Implementation has proceeded consistent with projected numbers of inspections with nearly all target units inspected in the first 4 years. Higher than expected inspection passage rates suggest that landlords have reduced lead hazards in rental housing affected by the law. Implementation of the lead law does not appear to have had a significant impact on the housing market.

Conclusions: Although many uncertainties remain, our analysis suggests that the lead law has had a positive impact on children’s health. Strong enforcement, support for community-based lead programs, and ongoing intergovernmental coordination will be necessary to maintain lead-safe housing in Rochester. Lessons learned from the Rochester experience may inform future local lead poisoning prevention policies in other communities.

Federal policies to reduce childhood lead poisoning, particularly bans on lead in paint and gasoline in the 1970s, resulted in drastic declines in the proportion of children with elevated blood lead (EBL; ≥ 10 μg/dL) from nearly 20% in 1990 to 1.6% in 2000 (Levin et al. 2008). Despite this progress, lead remains a critical environmental health hazard for many low-income children who live in housing built before 1978, when lead paint was banned for residential use in the United States. To address these remaining problems, some states have adopted additional policies to reduce lead poisoning. Most state policies focus on screening and management of children with EBL, but several promote housing-based primary prevention (Breysse et al. 2007; Brown et al. 2001). In addition to these state laws, a small number of cities have longstanding local lead laws aimed at housing-based primary prevention; in fact, several local laws preceded federal lead legislation (Freudenberg and Golub 1987).

Recognition that some communities continue to suffer disproportionately from lead and increased appreciation for the negative health effects of low-level lead poisoning sparked renewed interest in local lead policies in recent years. Rochester, New York, is one such community. In 2000, the proportion of children with EBL was substantially higher among screened children in high-risk neighborhoods in Rochester than in the state of New York or the United States as a whole; in 12 extreme-risk census tracts, > 35% of screened children were identified as having EBL (Boyce and Hood 2002).

Rochester is typical of cities in which the proportion of screened children with EBL far exceeds the national average. The vast majority of Rochester’s housing stock was built before 1978, with 87% constructed before 1950, when the highest amounts of lead in paint were used (Boyce and Hood 2002). Because of economic conditions in the city, many of these properties are now low-income rental units. Citywide, most housing units are rentals; in some neighborhoods, rental rates exceed 85%. National housing research has established that rental units are more likely to contain lead hazards than are owner-occupied units (Jacobs et al. 2002; Lanphear et al. 1998a).

Research conducted in Rochester in the 1990s highlighted both the high prevalence of EBL children and the developmental effects of even low levels of lead (Jones et al. 2009). The implications of the high prevalence of childhood EBL for children’s education and welfare caught the attention of child advocates in Rochester. These advocates, including educators, researchers, community groups, health care providers, and many others, formed the Coalition to Prevent Lead Poisoning (CPLP) in 2000 (Korfmacher 2008; Stoss 2005). CPLP quickly focused on the goal of passing a local policy to reduce lead hazards in high-risk housing. Although a wide range of stakeholders agreed on the importance of protecting children from lead hazards, many concerns were raised about the costs of lead hazard control, the most effective ways to protect children, the city’s financial and technical ability to implement lead hazard inspections, and the potential impact on Rochester’s already weak housing market. After several years of analysis, public debate, and policy advocacy, in December 2005, the Rochester city council passed an amendment to its housing code that requires lead inspections of rental properties built before 1978 (City of Rochester 2005a).

More than a dozen municipalities in the United States have recently enacted or amended local lead laws. Local lead laws are widely viewed as a promising approach to targeting the remaining gaps in childhood lead poisoning. However, no comprehensive evaluation has been conducted of the impacts of these laws. In this paper, we analyzed the impacts of Rochester’s lead law, using several existing sources of health and housing information, and provide recommendations for future local lead policies and research.

Housing-based lead hazards are the primary—but not the only—source of lead exposure among children in Rochester. Although the purpose of the lead law was to decrease the number of children with EBL in Rochester, local housing policy is only one of many factors affecting the number of children identified as having EBL, including housing markets, landlord and tenant knowledge, nonhousing lead sources, screening rates, and population dynamics. In addition, Rochester’s lead law targets only pre-1978 rental (not owner-occupied) housing. Therefore, rather than simply analyzing changes in EBL data since passage of the lead law, we evaluated the potential impacts of the lead law on factors identified by stakeholders in the policy process as key to determining the lead safety of rental housing. These factors include the effectiveness of the city’s inspections, the effects of the law on landlords’ maintenance practices, and impacts on the housing market.

## Rochester’s Lead Law

Rochester’s lead law targets high-risk housing to cost-effectively control lead hazards before children are poisoned (Korfmacher 2008). The policy integrates a visual inspection for deteriorated paint into the existing certificate of occupancy inspection system for pre-1978 rental housing. Properties found to have deteriorated paint in excess of HUD’s *de minimis* level or bare soil within 3 ft of the house fail the visual inspection. HUD standards allow for deteriorated paint below a *de minimis* level of 20 ft^2^ on any exterior surface, 2 ft^2^ in any interior room, or 10% of any component (such as a window sill) (City of Rochester 2005a). The soil provision was focused on the dripline area close to the house where lead paint scraped from the siding was most likely to result in elevated soil lead levels. In areas the city designates as high-risk based on past EBL data, units that pass the visual inspection must also pass a dust-wipe test based on federal standards. This provision was included as a quality control check on the city’s visual inspections and also because research has shown that units that pass visual tests for intact paint frequently contain invisible lead dust hazards (Breysse et al. 2007).

Under the lead law, property owners must correct any identified lead hazard violations before receiving a certificate of occupancy. To reduce compliance costs, the law allows owners or workers who have training in lead-safe work practices to complete repair work, rather than requiring U.S. Environmental Protection Agency (EPA)–certified abatement workers. The law allows the use of interim controls to address hazards. Interim controls such as repainting are less expensive than full lead abatement (permanent encapsulation or removal of lead). Although housing research has found interim controls to be effective in protecting children, homes treated in this way must be monitored so that the temporary controls remain intact (Dixon et al. 2005). In Rochester, this ongoing monitoring is accomplished through required periodic inspections of rental property. When the repair work is completed, those properties that were cited for an interior violation must pass a third-party clearance test to confirm that the lead hazards were addressed. These clearance tests must be conducted by U.S. EPA–certified inspectors using U.S. Department of Housing and Urban Development (HUD) clearance protocols (HUD 2004b).

The Rochester city council passed three accompanying resolutions to the lead law prioritizing inspections in target areas (Resolution 2005-23; City of Rochester 2005b), encouraging public education and establishing a citizen advisory group to inform implementation (Resolution 2005-24; City of Rochester 2005c), and requesting that the city establish a voluntary program for owner occupants (Resolution 2005-25; City of Rochester 2005d).

Key community goals were to inspect all rental properties by 2010 (the federally adopted target date for ending childhood lead poisoning) and to target initial inspections at the riskiest properties. Several features of the law target properties that are most likely to have lead hazards. First, a tenant complaint provision allows residents to request a free inspection by the city at any time. Second, homes of many families on public housing assistance from the county Temporary Assistance for Needy Families received more frequent inspections through the Quality Home Inspection program. Third, high-risk areas are targeted first. These areas were identified based on historical health department blood lead screening data. The lead law defined the high-risk area as those census block groups that cumulatively encompass an area in which no fewer than 90% of the units identified by the county health department for inspections in conjunction with its elevated blood lead–level inspections for the period of the preceding 5 years are located. These targeting strategies were implemented because research in other cities had suggested that lead poisoning can be efficiently prevented by focusing resources on the highest-risk neighborhoods (Haley and Talbot 2004; Meyer et al. 2005; Sargent et al. 1997).

## Methods

This analysis builds on an evaluation of the first 2 years of implementation of Rochester’s lead law that was conducted by the Center for Governmental Research (CGR) (Boyce et al. 2008). Resources for policy evaluation are generally scarce, particularly at the local level. In this case, however, the widespread community involvement in getting the lead law passed resulted in a commitment by Greater Rochester Health Foundation to support the analysis by CGR. CGR partnered with the City of Rochester, the Monroe County Department of Public Health (health department), the National Center for Healthy Housing (NCHH), and the Environmental Health Sciences Center of the University of Rochester to synthesize city inspection data, survey and conduct focus groups with landlords affected by the law, and analyze health department data on children’s blood lead levels. The University of Rochester Research Subjects Review Board (RSRB00033720) reviewed the use of human subjects data for this article and determined it to be exempt.

The CGR report and additional analyses conducted for this manuscript relied on three primary sources of data. The first was publicly available city housing inspections data, including the number and results of visual inspections, dust-wipe inspections, and exterior inspections. City housing inspectors trained in using the HUD Visual Assessment protocol (24 CFR Part 35; HUD 2004a) conducted visual assessments (City of Rochester 2005a). U.S. EPA–certified city lead inspectors took dust-wipe samples using HUD clearance protocols and analysis standards (HUD 2004b).

Second, we obtained blood lead data under a memorandum of agreement with the Monroe County Department of Public Health. The blood lead database of the county health department comprises blood lead results from all children tested under the lead screening law of New York State, which requires blood lead testing of all children at ages 1 and 2 years. Although the county health department does not calculate testing rates for the city, the number of children tested in Monroe County between 2004 and 2009 fluctuated by around 10% (between 13,624 and 14,917; no consistent trend). For the purpose of its analysis, CGR geocoded blood lead results to determine the number of EBL children who lived in the city during 12-month periods before and after implementation.

Third, CGR conducted two landlord focus groups and a telephone survey of 200 landlords. CGR surveyed by telephone a random sample of landlords drawn from the city’s list of all owners of two-unit (duplex) pre-1978 rental properties that were inspected during the first year after the law was implemented. Duplexes were chosen to maximize comparability of landlords’ experiences. The sample was restricted to owners of two-unit properties to limit variation in the size, housing characteristics, and value of the properties owned by the landlords included in the sample. Of the 373 landlords who were reached by phone, 200 completed the survey, for a response rate of 54% (landlords on the list were called in random order until 200 responses were obtained). City and county staff involved in lead programs also provided qualitative information about implementation. CGR recruited six landlords for the focus groups through local housing agencies. These focus groups explored landlords’ experiences with and perceptions about implementation of the lead law. Results were recorded, transcribed, and coded for common themes, which were integrated with survey results in the CGR analysis. Further information on methodology is provided in the CGR report (Boyce et al. 2008).

Publicly available inspection data from the City of Rochester and a data-sharing agreement with the health department made it possible to extend parts of this analysis through the third and fourth years of implementation (2008–2009 and 2009–2010). Where appropriate, we derived *p*-values for differences between time periods using chi-square tests. We used the Breslow–Day test for homogeneity of odds ratios using SAS version 9.2 (SAS Institute Inc., Cary, NC).

In 2010, the New York State comptroller’s office completed an audit of local lead control programs that provided additional evaluation of the lead law and its interactions with other local lead poisoning prevention efforts (Office of the New York State Comptroller 2010). Finally, the roles of the authors as participant observers (K.S.K. as a member of CPLP, M.A. as a member of CGR staff, and R.M. as Executive Director of the NCHH) provided ongoing access to the community, private, and government groups involved in the policy development and implementation process. Our findings are based on analysis of health department blood lead and city inspection databases and observational, qualitative (focus group), and quantitative (survey) data.

Our analysis focuses on the three major areas of concern that were debated before the law was passed: the inspection process of the city, the effectiveness of the law in protecting children from housing-based lead hazards, and the impacts on the housing market. We conclude with recommendations for future research and lessons learned for other communities interested in developing lead policies.

## Results

*Implementation of the law: city housing inspections.* Before the law was passed, debate about the proposed inspection process centered around two key issues: the cost of the lead inspections, and whether the city inspectors had the capacity to inspect all high-risk rental properties by 2010. To implement the lead law, the city hired and trained four new lead inspectors whose primary responsibility was to perform dust-wipe tests in high-risk units that passed a visual inspection for deteriorated paint. These inspectors, additional administrative needs, and analysis of lead dust wipes cost approximately $600,000 per year.

During discussions of the impacts of the lead law, city staff had estimated that they would inspect approximately 16,500 units each year. A total of 58,177 interior visual inspections were conducted in the first 4 years (Table 1)—within 15% of the number predicted by city staff before the law was adopted. As a result, the city was able to inspect nearly all pre-1978 rental units during the first 4 years of implementation.

**Table 1:** Interior inspections for deteriorated paint (visual inspections).

Inspection results	Year 1 1 July 2006–30 June 2007	Year 2 1 July 2007–30 June 2008	Year 3 1 July 2008–30 June 2009	Year 4 1 July 2009–30 June 2010	Total
No. of units inspected for deteriorated interior paint		16,449		11,607		13,355		16,766		58,177
No. (%) of units failing deteriorated interior paint inspection		958 (6)		1,380 (12)		699 (5)		684 (4)		3,721 (6)
No. (%) of units passing interior paint inspection		15,491 (94)		10,227 (88)		12,656 (95)		16,082 (96)		54,456 (94)
Years 1 through 4 after lead law implementation (1 July 2006–30 June 2010). Data from City of Rochester annual Lead Paint Poisoning Prevention Ordinance Inspection Review reports (City of Rochester 2005a).										

During the first 4 years of implementation, 94% of inspected properties passed the interior visual inspections (Table 1). This passing rate was much higher than had been anticipated based on prior lead assessments in high-risk areas. For example, in 2004, a community-based project that conducted full risk assessments in 70 homes in a high-risk neighborhood in Rochester found deteriorated leaded paint, lead in soil, or lead dust hazards in 95% of units (Korfmacher 2008; O’Fallon and Dearry 2002). As a result of this survey, as well as observations by city and county inspectors, CPLP members expected that Rochester would have much higher rates of housing with deteriorated paint than the national average. The National Survey of Lead and Allergens in Housing, a nationally representative, random sample of 831 housing units surveyed between 1998 and 2000, found only 14% had significantly deteriorated paint (Jacobs et al. 2002). Given the prior expectations, CPLP members were surprised that the actual visual inspection passing rate was higher than those in the national survey.

The national survey also indicated that lead in dust or soil lead hazards may exist in a significant number of units that pass a visual inspection (Jacobs et al. 2002). To address this risk, the Rochester law requires that units in high-risk areas that pass an interior visual inspection for deteriorated paint also pass a dust-wipe test. During the first 4 years, 20,555 units were referred for dust-wipe inspections (Table 2). A lower percentage of referred units received dust-wipe inspections in the first 2 years than in the second 2 years of implementation (77% vs. 88%; *p* < 0.001). According to city officials, the initially slow rate of dust testing was due to administrative challenges in scheduling follow-up visits with landlords. The increased proportion of referred units receiving dust-wipe testing in later years reflects increased efficiency of implementation as city inspectors and landlords adjusted to the requirements of the new law. Because of phased-in implementation (to keep the inspectors’ workload manageable, the city defined a smaller initial high-risk area in year 1), the total number of properties eligible for dust-wipe referrals increased in year 2 when the high-risk area was expanded to its full extent. This explains why the total number of units referred for dust wipes increased (from 3,850 to 5,778) in the second year.

**Table 2:** Dust-wipe tests in units passing visual inspections in high-risk areas.

Inspection results	Year 1 1 July 2006– 30 June 2007	Year 2 1 July 2007– 30 June 2008	Total for Years 1 and 2	Year 3 1 July 2008– 30 June 2009	Year 4 1 July 2009– 30 June 2010	Total for Years 3 and 4	Total
No. of units referred for dust-wipe test*a*		3,850		5,778		9,628		5,320		5,607		10,927		20,555
No. (%) of referred units that received dust-wipe test		2,850 (74)		4,606 (80)		7,456 (77)		4,654 (87)		4,940 (88)		9,594 (88)		17,050 (83)
No. (%) of units that passed dust-wipe test		2,420 (85)		3,936 (85)		6,356 (85)		4,242 (91)		4,518 (91)		8,760 (91)		15,116 (89)
No. of units cleared after failing dust-wipe test*b*		251		683		934		446		541		987		1,921
**a**Units located in high-risk areas that pass an interior inspection for deteriorated paint were referred for dust-wipe testing. **b**After failing the initial dust-wipe test, some units do not clear (complete required repairs and pass subsequent dust-wipe test) until a later year.														

Of the 17,050 units that actually received dust-wipe tests during the first 4 years of implementation, 89% passed. This passage rate exceeded predictions based on the National Survey of Lead and Allergens in Housing, in which only 67% of units with intact paint had no interior dust hazards (Clickner et al. 2001). Dust-wipe test passing rates significantly increased from years 1 and 2 to years 3 and 4 (85% vs. 91%; *p* < 0.001), which may indicate, as suggested by landlord survey and focus group participants, that property owners learned how to repair hazards effectively and to do these repairs before inspections as they gained experience complying with the law. Nonetheless, the dust-wipe testing identified almost 500 units each year (1,921 over the first 4 years of implementation) (Table 2) that had hazardous levels of lead in household dust, despite passing a visual inspection.

Because the city records exterior inspections by property (each of which may consist of multiple units), exterior violations data were reported separately from interior violations (Table 3). A total of 40,889 exterior inspections were conducted in the first 4 years; these inspections resulted in 5,637 citations (86% passing rate) for deteriorated paint or bare soil within 3 ft of the house. No clear pattern of change was observed in passing rates of exterior inspections over time.

**Table 3:** Visual inspections for exterior lead hazards (deteriorated paint or bare soil).

Inspection results	Year 1 1 July 2006–30 June 2007	Year 2 1 July 2007–30 June 2008	Year 3 1 July 2008–30 June 2009	Year 4 1 July 2009–30 June 2010	Total
No. of buildings inspected for exterior lead hazards		10,548		10,619		8,612		11,110		40,889
No. (%) of buildings that passed exterior lead hazards inspection		8,588 (81)		9,391 (88)		7,339 (85)		9,934 (89)		35,252 (86)

*Effectiveness of the law: impacts on children’s blood lead levels.* Evaluating the impact of the law on children’s lead levels is complex. One approach is to track changes in the extent of lead hazards in children’s homes. Ideally, one might conduct independent risk assessments in homes that had passed the city inspection to determine whether the environments were indeed lead safe. Because past research has correlated dust lead levels with blood lead levels, low dust lead levels in homes that passed the city inspection would suggest that the law is effectively protecting children’s health (Lanphear et al. 1996, 1998b). However, conducting independent risk assessments of inspected units was prohibitively expensive and logistically challenging—landlords were unlikely to grant access to their properties for a nonmandatory inspection. Instead, CGR compared results of city lead inspections with the results of subsequent inspections conducted by the county health department as part of case management for an EBL child. Although this was uncommon, in several cases county inspectors found hazards in properties that recently had passed city inspections. City and county staff reviewed the specific lead hazards in these cases and found that many of the hazards identified by county inspections were below the *de minimis* standard of the city lead law for lead violations. Nonetheless, city staff plan to use these findings for ongoing training of city inspectors. Both the CGR report and the state comptroller’s audit recommend that the city inspections office conduct annual cross-comparisons with health department inspections to identify any patterns of hazards in the homes of EBL children that were not detected during city lead inspections, or treatments that failed to eliminate hazards to children who were subsequently identified as having EBL.

A second approach to assess the effect of the lead law on children’s health is to examine trends in blood lead levels before and after implementation of the law. Before the passage of the law, some CPLP members speculated that a flurry of home renovations (not using lead-safe work practices) would be conducted in an attempt to comply with the law and would generate additional lead-laden dust. Thus, there were concerns that initial implementation might increase children’s lead exposures. It is reassuring to note that the prevalence of EBL among children tested in Rochester declined from 8.3% 2 years before implementation to 4.4% 2 years after implementation (*p* = 0.027) (Table 4). Although it is possible that renovation-related exposures increased during this time period, but were masked by background declines in EBL, such an effect was not reflected in the overall proportion of children with EBL.

**Table 4:** Children’s blood lead results, City of Rochester, July 2004–June 2008.*^a^*

Preimplementation of lead law			Postimplementation of lead law		
Level of blood lead	Year –2 1 July 2004–30 June 2005	Year –1 1 July 2005–30 June 2006	Year 1 1 July 2006–30 June 2007	Year 2 1 July 2007–30 June 2008
No. of children screened		7,256		7,420		7,146		6,528
Mean BLL (µg/dL)		4.73		4.21		4.00		3.73
Median BLL (µg/dL)		4.00		3.00		3.00		3.00
No. of children with BLL ≥ 10 µg/dL		604		490		403		284
Percentage of children with BLL ≥ 10 µg/dL		8.3		6.6		5.6		4.4
BLL, blood lead level. **a**These results are based on health department BLL data from the 2 years before and 2 years after implementation of the lead law (see Boyce et al. 2008).								

CGR pursued a third approach based on the fact that Rochester’s lead law applied only to pre-1978 rental housing. In the 2 years before the law’s implementation, 79% (year –2, 1 July 2004–30 June 2005) and 71% (year –1, 1 July 2005–30 June 2005) of positive properties (those the county found to have lead hazards in the course of investigating the housing of an EBL child with blood lead levels > 15 µg/dL) were rentals (Table 5), higher than the proportion of homes citywide that were rentals (56% in 2006). This is consistent with research findings that rental housing tends to have more lead hazards than owner-occupied housing (Jacobs et al. 2002). Because the lead law affects only rental housing, it was expected that the proportion of positive properties that were owner-occupied properties would increase after passage of the lead law. However, the proportion of positive properties that were owner occupied did not change significantly when comparing the 2 years before implementation with the 3 years after (24% vs. 21%; *p* = 0.480).

**Table 5:** Positive properties in City of Rochester, by ownership status, July 2004–June 2009 [*n* (%)].*^a^*

Property type	Year –2 1 July 2004– 30 June 2005	Year –1 1 July 2005– 30 June 2006	Total for Years –2 and –1	Year 1 1 July 2006– 30 June 2007	Year 2 1 July 2007– 30 June 2008	Year 3 1 July 2008– 30 June 2009	Total for Years 1 to 3
Positive*a*		114		89		203		132		114		110		356
Positive and ownership status could be determined		108		88		196		129		104		97		330
Owner occupied		23 (21)		25 (28)		48 (24)		21 (16)		27 (26)		24 (25)		72 (21)
Investor owned*b*		85 (79)		63 (71)		148 (76)		108 (84)		77 (74)		73 (75)		258 (79)
**a**Units found to have lead hazards in the course of the health department’s environmental investigation of lead in the home environment of a child with a blood lead level ≥ 15 µg/dL. **b**Rental properties.														

Another way to examine this effect is to see whether the proportion of EBL children who lived in rental housing declined relative to the proportion of EBL children who lived in owner-occupied homes after implementation of the lead law. Because of data limitations of the county health department’s screening database, CGR was unable to determine the ownership status of all units in which a child with an EBL resides. Therefore, we used a case–control approach to compare a random sample, taken from screening data, of 100 EBL cases and 100 non-EBL cases for each year of implementation. We calculated odds ratios comparing EBL among children living in a rental unit versus an owner-occupied unit. The resulting analysis showed that odds ratios after implementation were lower than in the 2 years before implementation. However, this change was not statistically significant [2.42, 95% confidence interval (CI): 1.69, 3.48 vs. 3.45, 95% CI: 2.07, 5.75; *p* = 0.276] (Table 6).

**Table 6:** Odds ratios of EBL children residing in rental property versus EBL children living in owned-occupied property, City of Rochester, July 2004–June 2009.

Preimplementation of lead law					Postimplementation of lead law						
Year –2 1 July 2004– 30 June 2005	Year –1 1 July 2005– 30 June 2006	Total for Years –1 and –2	Year 1 1 July 2006– 30 June 2007	Year 2 1 July 2007– 30 June 2008	Year 3 1 July 2008– 30 June 2009	Total for Years 1 to 3
Odds ratio (95% CI)		3.00 (1.43, 6.29)		3.93 (1.93, 8.00)		3.45 (2.07, 5.75)		3.49 (1.85, 6.59)		1.92 (1.05, 3.51)		2.15 (1.34, 4.08)		2.42 (1.69, 3.48)


*Costs of compliance: impacts on rental housing market.* During discussions leading up to passage of the lead law, one of the most significant areas of concern was the predicted economic impact of the lead law on the housing market in Rochester. Because of low property values and narrow profit margins in Rochester’s rental housing, landlord groups asserted that the additional costs of complying with the lead law would cause widespread abandonment of rental properties. Although it is difficult to separate the impacts of the lead law from other ongoing changes in the housing market, the CGR landlord survey and focus group results suggest that the lead law has not resulted in significant additional costs to landlords nor disruption of the rental housing market.

Results from the CGR telephone survey of landlords of duplex properties contributed to understanding of the law’s impacts on Rochester’s rental housing market. Among the 183 respondents who provided cost of compliance information, 34% said they spent nothing, 37% spent < $1,000, and 30% spent > $1,000 (Table 7). The mean per unit cost of repairs was $1,726 (median, $300). Notably, owners of higher-value duplex properties (> $40,000) spent less on repairs than did owners of lower-value duplex properties; over half of the higher-value properties required no or minimal repairs to comply with the new law (55% spent < $250; median, $120).

**Table 7:** Cost of repairs to comply with lead law [*n* (%)].

Repair costs	All respondents	Properties valued < $40,000	Properties valued ≥ $40,000
No. of duplex units		183 (100)		89 (100)		94 (100)
Total cost of repairs						
$0		63 (34)		21 (24)		42 (45)
$1–$250		25 (14)		16 (18)		9 (10)
$251–$1,000		42 (23)		24 (27)		18 (19)
$1,001–$2,500		25 (14)		15 (17)		10 (11)
$2,501–$5,000		16 (9)		7 (8)		9 (10)
> $5,001		12 (7)		6 (7)		6 (6)
Median cost		$300		$400		$120
Mean cost		$1,726		$2,265		$1,211
Data from Boyce et al. (2008). Data were taken from survey of owners of duplexes inspected in the prior year; respondents were asked to estimate costs for just those repairs made because of the lead law. These responses include both anticipatory repairs (prior to the inspection) and those conducted to correct a violation cited under the lead law.						

Respondents were not asked to distinguish whether they made repairs in anticipation of an inspection or to comply with a citation under the law. However, the fact that so many properties passed visual inspections suggests that most owners undertook necessary repairs before their inspection took place. Although this may have contributed to the higher-than-expected passing rates, one possible drawback to property owners prepping their units before inspection is that workers performing repairs may not have been trained in lead-safe work practices, as required when a unit is cited under the lead law. The U.S. EPA Renovation, Repair and Painting rule (RRP), implemented in April 2010, requires training of all paid workers (including landlords) who disturb paint in pre-1978 dwellings (U.S. EPA 2008). Before implementation of the RRP, there was concern that repair work conducted without lead-safe work practices would result in units that would appear safe, yet have extremely high levels of lead in remaining dust (Breysse et al. 2007). However, the 88% passing rate for dust-wipe tests conducted in units that passed the visual inspection suggests that preinspection renovation work generally resulted in lead-safe units. Both of these factors suggest that landlords used lead-safe work practices when making repairs.

CGR’s landlord focus groups affirmed that property owners did not find complying with the law prohibitively costly. Most violations were addressed by paint repair and cleaning, although some owners chose to conduct more extensive repairs, like window replacement. City inspectors provided property owners cited under the lead law with information on the city and county HUD-funded Lead Hazard Control grant programs in Rochester. Between 2003 and 2009, these and other local grant programs allocated $45 million to make > 1,200 units in Rochester lead safe (Office of the New York State Comptroller 2010). Although this was a small percentage of all the pre-1978 housing units in the city, these grant programs provided a resource to owners who needed financial assistance to undertake major repairs.

Before passage of the lead law, property owners also expressed concern about the cost of clearance testing. Under the lead law, owners must hire a private firm to conduct visual and dust-wipe testing after repairs are completed to clear the violation in a cited property. Initial estimates were that clearance testing would cost around $300 per unit. However, city staff reported that the average costs for clearance dropped to < $150 after implementation of the law as more firms became certified to conduct this testing and competition increased.

A separate concern raised by officials from the county Department of Human Services (DHS) was the impact that the law might have on demand for emergency housing. DHS provides emergency housing for a variety of crisis situations, including health and safety hazards. To examine this issue, CGR requested emergency placement data from DHS for 1 year before the law went into effect and for 2 years after. The number of emergency placements for lead contamination remained very low (between 3 and 13 of the approximately 9,000 annual emergency housing requests). According to city and county staff, the impacts of the law on demand for emergency placement may have been limited because *a*) many units are inspected and repaired while vacant; *b*) the majority of cited units can be repaired without relocating residents; and *c*) tenants and landlords prefer private relocation to emergency placement (i.e., staying with friends or relatives or going to a hotel). Overall, it appears that the lead law has not had the negative effects that some landlords predicted it would have on rental housing in Rochester. Indeed, the CGR focus group participants were “enthusiastic about the law and felt that it will help children in the City” (Boyce et al. 2008).

## Discussion

Rochester used the best available medical and housing research, combined with local data, to design a cost-effective, targeted lead law. However, because many of the law’s features were novel, there were many uncertainties about its potential consequences. Using diverse sources of available information, we explored a variety of perspectives on the law’s impacts. Our analysis was limited by the nature, extent, and quality of available data, including the health department blood lead screening database and the city inspections records. The new data collection efforts conducted by CGR (the landlord survey and focus groups) were limited by their small sample size. Nonetheless, our analysis of the available data suggests several lessons for other communities considering local lead laws.

First, it appears that Rochester’s system is reducing lead hazards in rental housing. Although the lead law protocol does not result in elimination of lead hazards, it does raise the bar for lead safety in the city’s highest risk housing. Because landlords can opt to use interim lead hazard controls instead of permanent lead hazard abatement, it is essential to continue regular inspections to monitor lead safety over time. The existence of higher-standard lead hazard control programs, such as the health department’s system of environmental investigations for EBL children and HUD lead hazard control grants for houses needing major repairs, provides the opportunity for ongoing quality control.

Second, the law contained several built-in provisions to monitor its effectiveness. For example, the requirement for dust-wipe testing in units that pass a visual inspection provides an objective source of quality control for the visual inspections. Because the law requires an annual implementation report, both city council and the public can track the number of inspections and passing rates over time.

Third, the design of the Rochester law requires ongoing collaboration among stakeholders. For example, as emphasized by City Council Resolution 2005-24 (City of Rochester 2005c), community education efforts are essential to make sure that tenants know *a*) their roles in maintaining lead-safe housing (e.g., cleaning, reporting damaged paint), *b*) their right to request inspection on demand, and *c*) ways to protect themselves from landlord retaliation. The availability of free lead-safe work training is also important to ensure that owners who do work on their properties do so safely. Financial resources to help landlords make repairs, to subsidize clearance costs, and to support the inspection program also support effective implementation. Continued community advocacy and commitment by local government leaders can help ensure that these resources remain available.

Many questions remain about the impacts of Rochester’s lead law, including

Effectiveness of inspection protocols: The fact that only 6% of Rochester properties failed visual inspections suggests that either property owners were making sure paint was in good repair before their inspections or that visual inspections were not identifying all deteriorated paint. The high dust-wipe passing rate suggests that the visual inspections were effective in identifying lead hazards; otherwise, more units would have failed the dust-wipe tests. Alternatively, the higher-than-expected dust-wipe passing rate calls into question the effectiveness of the dust-wipe tests themselves. Dust-wipe protocols are standardized; however, they may miss hazards. For example, dust-wipe results may be skewed by taking samples only in well-cleaned areas. Rochester’s high dust-wipe test passage rate suggests that either landlords were successfully addressing lead dust hazards before inspection or that the inspections were not fully effective in identifying hazards. A systematic study of the city’s dust-wipe tests could determine whether they are conducted effectively and consistently.
Long-term costs and effectiveness: The lead law allows interim controls of lead hazards, which by definition do not permanently address lead hazards without ongoing maintenance. As properties come up for reinspection, it is important to monitor whether interim controls have been maintained and whether landlords have found it cost effective to implement more permanent measures. Comparing inspection results on properties with their prior inspection records and repeating the landlord survey could shed light on these questions.
Impacts on high-risk families: This evaluation did not directly address how the lead law has affected families at risk of lead poisoning. Although there are no data suggesting that the law is causing homelessness (as indicated by the low rate of lead-related emergency housing placements), it is possible the law has made it more difficult for families to find housing. It is also not known how well tenants understand their rights under the law and their role in maintaining lead safety over time.
Long-term effects on blood lead levels: Inspection results, landlord feedback, and qualitative observations from staff at implementing agencies suggest that the lead law has contributed to declining blood lead levels among children living in rental housing, but other factors (e.g., ongoing demolition of high-risk housing, grant programs, public education) have likely contributed also. It is important to continue to monitor inspection results, blood lead levels, and the ownership status of units where EBL children reside. It might also be revealing to compare EBL rates over time in high-risk census tracts in Rochester with those in similar cities that do not have a lead law.

Rochester’s lead law embodies several new approaches to reducing lead hazards in housing that were designed to be cost effective. Many other promising proposals were considered and rejected during the policy debate that may be adopted in other cities. As other communities experiment with lead policy innovations, they should evaluate and share their experiences to contribute to our national understanding of how to design effective local lead policies.

## Conclusions

Evaluation is a critical part of the policy process (Hu and Brown 2003). However, evaluation of local policy impacts is often neglected because of financial limitations, available staff time, data constraints, and technical complexity. In the case of the Rochester lead law, the support of a local foundation and a partnership of private consultants, academics, government, and community groups provided valuable insights into the initial impacts of this groundbreaking local law.

Implementation has proceeded much as predicted, with nearly all pre-1978 rental units inspected by the end of 2010. The resources required for implementation have been similar to what was anticipated by the city. Although city government has strongly supported this program, there are concerns that implementation costs may not be sustainable because of anticipated future budget constraints.

Despite assertions by landlord advocates that adopting the lead law would result in massive abandonment of rental properties, the lead law does not appear to have had a significant impact on the rental housing market. Although a comprehensive analysis of changes in the housing market attributable to the lead law was beyond the scope of our study, publicly available housing data did not indicate a marked change.

The evaluation also suggests that the lead law has contributed to continued declines in children’s blood lead levels by decreasing the extent of lead hazards in pre-1978 rental housing; however, additional information on the proportion of EBL children living in targeted housing is needed to confirm this finding. The fact that 94% of units passed visual inspections and that 89% of tested units passed dust-wipe inspections during the first 4 years—both much higher passing rates than predicted based on prior local and national studies—is perhaps the strongest indicator that the lead safety of rental housing has improved since passage of the Rochester lead law. The high visual inspection passing rate may indicate that landlords are performing repairs to reduce lead hazards before inspections. In addition, the unexpectedly high passing rates for dust wipes suggest that these repairs are being done using lead-safe work practices. Alternatively, the dust-wipe tests may not be identifying all hazards. Therefore, it is essential to conduct ongoing quality assurance inspections to make sure dust-wipes tests are being carried out effectively. The trends in children’s blood lead levels do not suggest that the law has created new hazards; additional analysis would be needed to confirm that the lead law is protecting children from being exposed to lead hazards over time.

Government, academic, private-sector, and community groups continue to communicate regularly about the lead law. These conversations allow stakeholders to jointly identify weaknesses, develop solutions, and prioritize approaches to new challenges. The implementation environment of state and federal policies, the housing market shifts, and implementation resources are constantly changing. Over time, therefore, Rochester’s collaborative process of evaluation may be a key to the long-term success of its new lead policy.

Local lead laws can help protect children from the lead that will remain in U.S. housing for decades to come. They are an important complement to federal and state programs and policies currently in place, particularly in high lead risk communities. However, our understanding of the most effective local approaches is in its infancy. Therefore, efforts to evaluate local policy innovations may be a key to sustaining declines in lead poisoning. Local governments and communities need pragmatic, sustainable systems to track progress over time, identify unintended consequences, and suggest opportunities for improvement in their policy innovations.
